# Language and cultural bias in AI: comparing the performance of large language models developed in different countries on Traditional Chinese Medicine highlights the need for localized models

**DOI:** 10.1186/s12967-024-05128-4

**Published:** 2024-03-29

**Authors:** Lingxuan Zhu, Weiming Mou, Yancheng Lai, Junda Lin, Peng Luo

**Affiliations:** 1 Department of Oncology, Zhujiang Hospital, Southern Medical University, 253 Industrial Avenue, Guangzhou, 510282 China; 2 Department of Urology, Shanghai General Hospital, Shanghai Jiao Tong University School of Medicine, 100 Haining Road, Hongkou District, Shanghai, China

To the Editor,

Large language models (LLMs) are AI systems trained on vast amounts of text data to understand human language and interact with humans in natural language. ChatGPT, a well-known example of LLMs developed by OpenAI, has demonstrated powerful capabilities across many domains, including passing various standardized medical qualification exams such as the United States Medical Licensing Examination (USMLE) [1]. Studies suggest its potential to enhance medical education and aid clinical decision-making [2]. Although ChatGPT can answer questions in multiple languages, it should be noted that, like many Western-developed LLMs, the vast majority of its training data is in English [3], and many studies on its capabilities in the medical field is based on a Western medical context. Traditional Chinese Medicine (TCM), rooted in millennia of Chinese wisdom, differs fundamentally from Western medicine. Due to the relatively small proportion of Chinese in the training corpus, models develpoed by Western countries, such as ChatGPT, may lack sufficient exposure to concepts and terminology from TCM, casting doubt on their application in TCM. Chinese companies have developed their own LLMs, such as Baidu’s Ernie Bot series, ZHIPU AI’s GLM-4, and Alibaba’s Qwen-max, which leveraged extensive Chinese language data in their training [4]. We compare the accuracy of LLMs developed by China and the West in answering TCM-related questions to explore the capabilities of LLMs in understanding and applying domain-specific knowledge across different languages and cultural backgrounds.

The National Medical Licensing Examination for Traditional Chinese Medicine (TCM) is the qualification entrance exam for TCM practitioners in China and consists of multiple-choice questions divided into four units. We utilized 140 questions from the first unit of the 2022 examination, which assesses fundamental TCM subjects such as Fundamentals of TCM, Diagnostics, Chinese Materia Medica, and Herbal Formulas. Questions related to Health Laws and Regulations were excluded as they are unrelated to TCM. Eight prominent LLMs were included in our study: four from Chinese companies (Ernie Bot [Baidu], Ernie Bot-4 [Baidu], Qwen-max [Alibaba], and GLM-4 [ZHIPU AI]) and four from western countries (ChatGPT-3.5 [OpenAI], ChatGPT-4 [OpenAI], Claude-2 [Anthropic], and Gemini-pro [Google]). To ensure models correctly identified the questions as TCM-related, we added “In traditional Chinese medicine theory” to the beginning of each question and submitted the questions and options together to the LLMs. Responses were collected on January 2, 2024 via API (default parameters). All interactions were conducted in Chinese. To mitigate the impact of inherent randomness in LLMs on the evaluation, each model generated three answers per question. Correctness was defined as answering questions correctly twice or more.

The performance of eight LLMs on the TCM exam is shown in Fig. 1A. The average accuracy of LLMs developed by Chinese companies (78.4%) was higher than that of those developed by Western companies (35.9%, Wilcoxon test, p < 0.05). Among the Chinese models, Qwen-max achieved the highest accuracy of 86.4%, followed by GLM-4 (80.7%) and Erine Bot-4 (78.6%). Most of the LLMs developed in China outperformed LLMs developed in the West (Fig. 1B. McNemar's test with Bonferroni correction). All Chinese models passed the exam with accuracy rates above 60%, whereas all western models failed. We further evaluated whether the models are better at answering questions from a particular subject, and the result showed no significant difference for most models. However, for Ernie Bot and Qwen-max, there was a significant difference when requiring at least 2 or 3 correct responses (Additional file 1: Table S1).

**Fig. 1:**
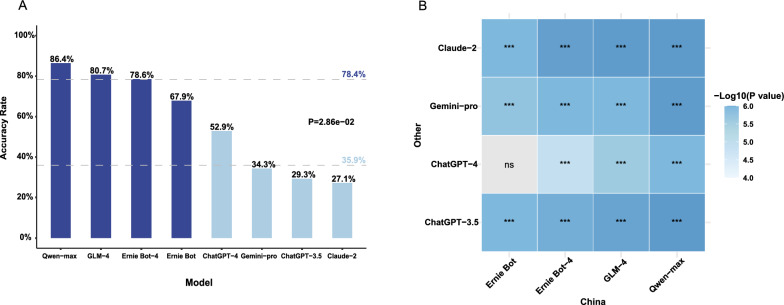
Performance of 8 Large Language Models (LLMs) on Traditional Chinese Medicine (TCM)-related Questions. **A** Accuracy rates of different models. Horizontal lines represent the accuracy rates of Chinese-developed and Western-developed LLMs on Traditional Chinese Medicine questions, respectively. P values were calculated using the Wilcoxon test to compare the accuracy rates of Chinese-developed and Western-developed LLMs on Traditional Chinese Medicine questions. **B** Heatmap showing the P values for comparisons of accuracy between each Chinese-developed LLM and each Western-developed LLM. P values were calculated using McNemar’s test with Bonferroni correction. ns, no significance. *P < 0.05, **P < 0.01, ***P < 0.001

Our study highlights the impact of language and cultural biases on LLMs’ performance in the context of TCM. The difference in performance may stem from Western LLMs being primarily trained on English datasets, lacking deep familiarity with Chinese culture, language nuances, and TCM concepts. This challenge extends beyond the Chinese context, as evidenced by ChatGPT-3.5's failure in the Japanese medical licensing exam [5]. To address these limitations, we suggest developing localized LLMs trained on local data or prioritizing multilingual and cross-cultural training data. In China, domestically developed LLMs can provide more accurate interpretations of medical knowledge based on the characteristics of the Chinese population and current clinical practice in China. Such models can also understand the linguistic nuances, idioms, colloquialisms, and cultural aspects of Chinese, offering services that better meet user needs within China's sociocultural context. Moreover, independently developed LLMs can improve data security and privacy protection while helping to preserve and transmit Chinese culture.

In conclusion, our exploration underscores the importance of developing LLMs tailored to specific languages and cultural contexts, particularly in the field of TCM. With continuous localization optimizations and advancements in AI, China's LLMs could make significant contributions across various fields, unlocking endless possibilities.

### Supplementary Information

**Additional file 1: Table S1.** Performance of Ernie Bot, Ernie Bot-4, Qwen-max, GLM-4, ChatGPT-3.5, ChatGPT-4, Claude-2 and Gemini-pro on National Medical Licensing Examination for Traditional Chinese Medicine (TCM). Statistical significance was assessed using Fisher’s exact test. If statistical differences were observed, subgroup analysis were performed using Fisher’s exact test with Bonferroni correction for multiple comparisons to evaluate the accuracy rate of one subject against the combined accuracy rate of the remaining three subjects within the same model.

## Data Availability

The datasets used and/or analysed during the current study are available from the corresponding author on reasonable request.

## Abbreviations

AI: Artificial Intelligence
LLM: Large language model
TCM: Traditional Chinese Medicine
USMLE: United States Medical Licensing Examination
