# Supplementary material for: Language and cultural bias in AI: comparing the performance of large language models developed in different countries on Traditional Chinese Medicine highlights the need for localized models
Source: J Transl Med. 2024 Mar 29;22:319. doi: 10.1186/s12967-024-05128-4 (PMC10981296; doi:10.1186/s12967-024-05128-4)
Supplement: Supplementary file 1 — Additional file 1: Table S1. Performance of Ernie Bot, Ernie Bot-4, Qwen-max, GLM-4, ChatGPT-3.5, ChatGPT-4, Claude-2 and Gemini-pro on National Medical Licensing Examination for Traditional Chinese Medicine (TCM). Statistical significance was assessed using Fisher’s exact test. If statistical differences were observed, subgroup analysis were performed using Fisher’s exact test with Bonferroni correction for multiple comparisons to evaluate the accuracy rate of one subject against the combined accuracy rate of the remaining three subjects within the same model. [file 12967_2024_5128_MOESM1_ESM.pdf]

Supplementary Table 1. Performance of Ernie Bot, Ernie Bot-4, Qwen-max, GLM-4, ChatGPT-3.5, ChatGPT-4, Claude-2 and Gemini-pro on National Medical Licensing Examination for Traditional Chinese Medicine (TCM). Statistical significance was assessed using Fisher's exact test. If statistical differences were observed, subgroup analysis were performed using Fisher's exact test with Bonferroni correction for multiple comparisons to evaluate the accuracy rate of one subject against the combined accuracy rate of the remaining three subjects within the same model.

Question Type	Number of questions	Correct Times ≥ 1		Correct Times ≥ 2		Correct Times = 3	
		Number of correct answers	P value	Number of correct answers	P value	Number of correct answers	P value
**Ernie Bot**			0.073		0.011		0.010
**Fundamentals of TCM**	42	25 (59.52%)		21 (50%)	0.020	16 (38.1%)	0.020
**Diagnostics of TCM**	33	26 (78.79%)		26 (78.79%)	0.564	24 (72.73%)	0.176
**Chinese Materia Medica**	34	29 (85.29%)		28 (82.35%)	0.224	23 (67.65%)	0.660
**Chinese Herbal Formulas**	31	22 (70.97%)		20 (64.52%)	1.000	16 (51.61%)	1.000
**Ernie Bot-4**			0.581		0.363		0.766
**Fundamentals of TCM**	42	37 (88.1%)		35 (83.33%)		30 (71.43%)	
**Diagnostics of TCM**	33	30 (90.91%)		24 (72.73%)		23 (69.7%)	
**Chinese Materia Medica**	34	31 (91.18%)		29 (85.29%)		26 (76.47%)	
**Chinese Herbal Formulas**	31	25 (80.65%)		22 (70.97%)		20 (64.52%)	
**Qwen-max**			0.475		0.163		0.038
**Fundamentals of TCM**	42	36 (85.71%)		32 (76.19%)		28 (66.67%)	0.032
**Diagnostics of TCM**	33	29 (87.88%)		29 (87.88%)		29 (87.88%)	1.000
**Chinese Materia Medica**	34	31 (91.18%)		31 (91.18%)		31 (91.18%)	0.512
**Chinese Herbal Formulas**	31	30 (96.77%)		29 (93.55%)		26 (83.87%)	1.000
**GLM-4**			0.309		0.361		0.360
**Fundamentals of TCM**	42	33 (78.57%)		32 (76.19%)		31 (73.81%)	
**Diagnostics of TCM**	33	31 (93.94%)		30 (90.91%)		29 (87.88%)	
**Chinese Materia Medica**	34	28 (82.35%)		27 (79.41%)		26 (76.47%)	
**Chinese Herbal Formulas**	31	26 (83.87%)		24 (77.42%)		22 (70.97%)	
**ChatGPT-3.5**			0.761		0.500		0.700
**Fundamentals of TCM**	42	18 (42.86%)		12 (28.57%)		9 (21.43%)	
**Diagnostics of TCM**	33	17 (51.52%)		13 (39.39%)		9 (27.27%)	
**Chinese Materia Medica**	34	16 (47.06%)		9 (26.47%)		6 (17.65%)	
**Chinese Herbal Formulas**	31	12 (38.71%)		7 (22.58%)		5 (16.13%)	
**ChatGPT-4**			0.304		0.549		0.159
**Fundamentals of TCM**	42	24 (57.14%)		21 (50%)		16 (38.1%)	
**Diagnostics of TCM**	33	25 (75.76%)		21 (63.64%)		19 (57.58%)	
**Chinese Materia Medica**	34	19 (55.88%)		16 (47.06%)		11 (32.35%)	
**Chinese Herbal Formulas**	31	19 (61.29%)		16 (51.61%)		11 (35.48%)	
**Claude-2**			0.948		0.318		0.166
**Fundamentals of TCM**	42	20 (47.62%)		10 (23.81%)		7 (16.67%)	
**Diagnostics of TCM**	33	18 (54.55%)		13 (39.39%)		11 (33.33%)	
**Chinese Materia Medica**	34	18 (52.94%)		9 (26.47%)		5 (14.71%)	
**Chinese Herbal Formulas**	31	16 (51.61%)		6 (19.35%)		4 (12.9%)	
**Gemini-pro**			0.297		0.172		0.140
**Fundamentals of TCM**	42	20 (47.62%)		10 (23.81%)		5 (11.9%)	
**Diagnostics of TCM**	33	21 (63.64%)		16 (48.48%)		11 (33.33%)	

**Chinese Materia Medica**	34	21 (61.76%)	12 (35.29%)	9 (26.47%)
**Chinese Herbal Formulas**	31	14 (45.16%)	10 (32.26%)	8 (25.81%)
